# From health to harm: Cross-sectional analysis of factors associated with orthorexia nervosa

**DOI:** 10.1016/j.nsa.2026.107011

**Published:** 2026-05-12

**Authors:** Annika M. Roth, Regina Vollmeyer, Silke Matura

**Affiliations:** a Goethe University Frankfurt, Department of Psychology, Educational Psychology, Theodor-W.-Adorno-Platz 6, Frankfurt am Main, 60323, Frankfurt, Germany; b Goethe University Frankfurt, University Hospital, Department of Psychiatry, Psychosomatic Medicine and Psychotherapy, Heinrich-Hoffmann-Str. 10, 60528, Frankfurt, Germany

## Abstract

The present study systematically examined factors associated with orthorexic tendencies, to provide a clearer understanding of this phenomenon. In a cross-sectional setting, data were collected using an online questionnaire including validated instruments for assessing orthorexic tendencies and related variables. Therefore, correlation measures and difference tests were used. Hierarchical multiple linear regression was performed to identify variables independently associated with orthorexic tendencies. A total of *n* = 1049 participants were included in data analyses. Individuals with a current or past mental disorder had significantly higher DOS scores than those without such a history (Hedges’ *g* = 0.55, *p* < .001). Physically active participants had significantly higher orthorexic tendencies (*g* = 0.29, *p* = .01). Significant correlations between DOS scores and BMI, *r* = −0.11, *p =* .002, training days, *r* = 0.2, *p* < .001, engagement with health and fitness content, *r* = 0.35, *p* < .001, eating disorder symptomatology, *r* = 0.62, *p* < .001, perfectionism, *r* = 0.43, *p* < .001, and self-esteem, *r* = −0.41, *p* < .001, were observed. In the multivariate regression model, eating disorder symptoms showed the strongest independent association with orthorexic tendencies. The overall model explained 57.7 % of the variance in orthorexic tendencies. In the present sample, orthorexic tendencies showed substantial overlap with eating disorder symptomatology, which emerged as the dominant correlate in the multivariate model. Overall, the findings support interpreting orthorexic tendencies primarily in relation to broader eating disorder psychopathology.

## Highlights

• Study with more than 1000 participants, broadly examining factors related to ON.
• Findings suggest that ON develops through psychological, social and behavioral factors.
• Study provides a framework for enhancing preventative measures.

## Teaser

Orthorexia Nervosa (ON), an obsessive preoccupation with healthy eating, is an emerging phenomenon in the field of eating-related psychopathology that may negatively affect quality of life. In this study, we systematically examined factors associated with orthorexic tendencies using an online questionnaire. Our research found that orthorexic tendencies were higher among individuals with a history of mental health issues. Those who exercised regularly or spent more time engaging with health and fitness content on social media also showed higher orthorexic tendencies. Additionally, perfectionism was significantly associated with orthorexic tendencies. Overall, however, orthorexic tendencies were most strongly related to eating disorder symptoms. A better understanding of these associations may help to improve early identification, prevention, and support.

## List of abbreviations

AMI: Achievement Motivation Inventory
ANOVA: Analysis of Variance
ASS: Anstrengungsskala
BFI-2: Big Five Inventory 2
BMI: Body Mass Index
BQHPA: Baecke Questionnaire of Habitual Physical Activity
CI: Confidence Interval
DKB-35: Dresden Body Image Questionnaire-35
DOS: Düsseldorf Orthorexia Scale
DSM-5: Statistical Manual of Mental Disorders, Fifth Edition
EDE-Q: Eating Disorder Examination-Questionnaire
FDR: False Discovery Rate
ICD-11: International Classification of Diseases, 11th Revision
M: Mean Value
MI: Multiple Imputation
MICE: Multiple Imputation by Chained Equations
OCD: Obsessive Compulsive Disorder
ON: Orthorexia Nervosa
RSES: Rosenberg Self-Esteem Scale
SD: Standard Deviation
STROBE: STrengthening the Reporting of OBservational studies in Epidemiology
VIF: Variance Inflation Factors

## 1 Background

Orthorexia nervosa (ON) is an emerging phenomenon in the field of eating-related psychopathology and was first described by Steven Bratman in the late 1990s as an obsessive preoccupation with healthy eating (Bratman, 1997). In his book *Health Food Junkies*, Bratman described both his own experiences and those of people around him who were in a constant struggle with their diets (Bratman and Knight, 2000). Derived from the Greek, the term is a compound of ὀρθός*”* (*orthos*, right) and *“ὄρεξις”* (*orexis*, appetite) and refers to the pursuit of the “right” appetite (Donini et al., 2022). While most people strive for healthy eating, in cases of ON healthy eating behavior becomes a compulsion (Bratman and Knight, 2000).

Orthorexic eating behavior has often been discussed in relation to established eating disorders, particularly anorexia nervosa (Atchison and Zickgraf, 2022), due to overlaps in rigid eating patterns and dietary control. In contrast to anorexia nervosa, where the main intention is body mass loss through calorie restriction, ON places emphasis on food quality (Dunn and Bratman, 2016). Although weight changes may occur, they are not the primary concern of individuals with orthorexic tendencies, who instead focus on achieving a sense of purity and health through their food choices (Brytek-Matera, 2012). This fixation can lead to social isolation as those suffering from ON often avoid situations where they have no control over their food. In extreme cases, this can be associated with malnutrition and severe weight loss or other health problems due to reduced food intake (Dunn and Bratman, 2016). Since the concept of ON was first used by Bratman to describe an obsessive fixation on the perceived correct nutrition (Bratman, 1997), numerous other attempts have been made to define ON and the related diagnostic criteria (e.g. Barthels, 2014; Dunn and Bratman, 2016; Vandereycken, 2011). In this context, some authors have suggested distinguishing between ON and “healthy orthorexia”, referring to a non-pathological interest in healthy eating (Barrada and Roncero, 2018; Hanras et al., 2024). However, this distinction remains debated, as diagnostic proposals define ON through distress and functional impairment (Dunn and Bratman, 2016), thereby challenging the notion of a “healthy” variant. Thus, the current literature does not provide a clear consensus regarding this differentiation. An important step toward greater conceptual clarity was taken in 2019, when the Orthorexia Nervosa Task Force proposed the first standardized definition (Cena et al., 2019). According to this definition, ON is described as a constant concern with healthy eating that leads to a strong preoccupation with nutrition, including a persistent mental occupation with one's own eating behavior, as well as stereotypical eating behaviors, e.g. rigid dietary rules (Cena et al., 2019).

Although ON has become a global phenomenon (Cena et al., 2019; Fang et al., 2023; Valente et al., 2019), it is not recognized as a distinct eating disorder in either the Diagnostic and Statistical Manual of Mental Disorders (DSM-5) (American Psychiatric Association, 2013) or the International Classification of Diseases (ICD-11) (BfArM, 2022), thus, the ongoing debate about whether ON should be included as a separate diagnosis in these classification systems remains (Niedzielski and Kaźmierczak-Wojtaś, 2021). Unsurprisingly, no standardized diagnostic criteria for ON currently exist, thus complicating the efforts to identify associated factors and treatment approaches. This, ultimately, has led to disagreements in scientific research by virtue of the different measurement tools that have been developed based on inconsistent diagnostic criteria (Donini et al., 2022). Without clear definitions and diagnostic criteria, studies – especially those investigating associated factors – are difficult to compare, leading to varying prevalence rates and an unclear research landscape (Niedzielski and Kaźmierczak-Wojtaś, 2021; Valente et al., 2019).

In response to this, in 2021 and 2022, members of the Orthorexia Nervosa Task Force developed a systematically-derived proposal for standardized diagnostic criteria and potential associated factors using the Delphi-method (Donini et al., 2022). Although the experts agreed on the relationship between ON and various proposed associated factors (e.g. sports, perfectionism, influence of social media, diet, study and career choices, history of other mental disorders or (psycho)-somatic problems), the evidence is not always consistent (Donini et al., 2022). While the state of research appears to be clear for some factors associated with orthorexic tendencies, such as vegan/vegetarian diets (Brytek-Matera, 2020; Dell’Osso et al., 2018) or perfectionism (Horovitz and Argyrides, 2023; McComb and Mills, 2019), it remains to be determined whether sports (Bóna et al., 2019; Strahler et al., 2018) and social media usage (Tarsitano et al., 2022; Turner and Lefevre, 2017) are actual factors associated with orthorexic tendencies (Donini et al., 2022). Constituting a milestone in the scientific debate on ON, the standardization of diagnostic characteristics (Donini et al., 2022) significantly enhanced the comparability of studies by reducing methodological inconsistencies and uncertainties. Using this valid and consistent fundament, future studies will now be able to identify, precisely and thoroughly, factors associated with ON.

Given the growing awareness in research and that ON is not merely a preference for healthy foods but a worrisome obsession that can severely impair the quality of life for those affected, the present study examines the associated factors presented by Donini et al. (2022) in light of their proposed diagnostic criteria and, hence, provides novel data to clarify the unclear research landscape as requested by Varga et al. (2013), McComb and Mills (2019) and Łucka et al. (2024). This may help gain a deeper understanding of the factors associated with the orthorexic eating behavior, thus contributing to the improvement of prevention and treatment approaches. At the same time, examining factors associated with orthorexic eating behavior may contribute to a better understanding of how orthorexic tendencies relate to other disorders, including anorexia nervosa, other eating disorders, and obsessive-compulsive disorder.

In the present study, we examined various factors associated with orthorexic tendencies that have been highlighted by the Orthorexia Nervosa Task Force (Donini et al., 2022), focusing specifically on (1) physical activity, including participation in competitive sports, (2) social media usage, especially engagement with health and fitness-related content, (3) diet, (4) psychopathology, including hypochondria, depressive symptoms and anxiety, (5) personality traits, especially perfectionism, (6) self-concept, including self-esteem and body acceptance, and (7) sociodemographic characteristics. These factors were selected because of their importance in the consensus statement of the ON Task Force and their investigation in previous empirical studies, as well as their relevance for understanding orthorexic tendencies. By focusing on this subset of factors, we aim to examine their associations with orthorexic tendencies in a cross-sectional design and help to address inconsistencies in the current literature.

## 2 Methods

### 2.1 Procedure and participants

The study was prepared in accordance with the STROBE (STrengthening the Reporting of OBservational studies in Epidemiology) Statement for cross-sectional studies (STROBE, 2025). Preregistration was carried out using AsPredicted.org (#191764, https://aspredicted.org/wm5h-vsgj.pdf). The study was approved by the ethics committee from Goethe-University Frankfurt, GER, under the number #2024-40.

Data collection was carried out anonymously using an online questionnaire with SoSciSurvey, with participants (from the general population) mainly recruited via social media (e.g. Instagram and Facebook). In addition, flyers with the survey link were distributed at universities, supermarkets, and local clubs. The online survey was available from September, 2024 until January, 2025. Data were collected, evaluated and stored in accordance with the GDPR (Art. 13 to 22).

At the beginning of the questionnaire, participants were informed in detail about the survey and its objective, that participation was completely voluntary, and that the data would be collected anonymously. Furthermore, participants were informed that they could skip individual questions that they did not wish to answer and that they could withdraw from the survey at any time. By ticking the box, *“I have taken note of the information on data protection and consent to the intended processing of my data”*, the informed consent of the participants was obtained. All participants completed the survey in a fixed order, beginning with questions on physical activity, and followed by social media usage, diet, psychopathology, physical health, personality traits, self-concept, motivation, and finally sociodemographic characteristics. Given that the literature suggests that gender does not significantly contribute to ON scores (Donini et al., 2022; McComb and Mills, 2019), all participants were pooled for statistical analyses. The survey took around 20 minutes to complete. Bot activity was checked before data analyses by excluding responses with unrealistic completion times. Ultimately, all participants who both lived in Germany and were over the age of 18 years were included in the data analyses. A total of five vouchers worth €50 each were randomly distributed among all participants who provided their email address in a separate survey.

Given the inconsistent evidence regarding factors associated with ON – such as the small effect size |*r*| = 0.1 for perfectionism reported by Hayles et al. (2017) compared to the moderate effect found by Novara et al. (2021) |*r*| = 0.37 – we conducted our a priori power analysis assuming small effect sizes (|*r*| = 0.1) across associated factors with an alpha level of 0.05 and a beta level of 0.8. This resulted in an estimated required sample size of *n* = 782.

### 2.2 Measures

#### 2.2.1 Physical activity

Physical activity was measured based on the Baecke Questionnaire of Habitual Physical Activity (BQHPA) (Baecke et al., 1982). The BQHPA is a questionnaire used to assess the extent of physical activity in the domains of work, sport, and leisure. The original version of the questionnaire included 16 different items. In the present study, we used the German version (Wagner and Singer, 2002) and only included the items on sport. The BQHPA has demonstrated acceptable psychometric properties in previous research, with test-retest reliabilities ranging from *r* = 0.74 to *r* = 0.88 across all subscales (Baecke et al., 1982). Furthermore, the German version shows good reliability and construct validity (Wagner and Singer, 2002), making it a widely used instrument. Firstly, the participants were asked whether they engaged in sports. If they chose “yes”, then this was followed by questions on whether they practiced competitive sports (yes/no), and which sport they practiced most frequently (open response). Next, we asked about the number of hours of training per week (open response) and the number of months of training per year. Thereby, a 5-level scale from 1 = *less than* *1 month* to 5 = *more than* *9 months per year* was used. The participants were then asked whether they engaged in another type of sport (yes/no). If “yes” was selected, the questions on the type of sport, number of hours training per week and the number of months of training per year were asked again. Subsequently, the participants were asked about their physical activity in days per week on a 7-point scale from 1 = *1 day* to 7 = *7 days* and the average duration of training (open response) in minutes (Martinovic et al., 2022a). Finally, the average training intensity was captured with the question, *“On average, how would you describe the intensity of your training over the last four weeks?”*. Ratings were based on the German *Anstrengungsskala* (ASS) from 1 = *not at all exhausting* to 10 = *so exhausting that I had to stop* (Büsch et al., 2022).

#### 2.2.2 Social media

Based on Turner and Lefevre (2017), the participants were first asked to provide information on how often they accessed various social media platforms with the following proposed platforms: Facebook, Instagram, Threads, YouTube, Pinterest, Snapchat, TikTok, X, and Tumblr. These platforms are all content-based and it is possible to add or follow other users. The number of entries was given on a 7-point scale from 1 = *less than once a month* to 7 = *several times a day*. It was possible to indicate that none of the proposed social networks were used. Participants were also asked to provide the amount of time they spent on each platform in minutes per day (Turner and Lefevre, 2017). Finally, engagement in health and fitness accounts on social media was measured using a scale by Scheiber et al. (2023). The scale consisted of three items (e.g. “*Health & fitness-related content on the social media channels I use is important to me”*) while answers were given using a 7-point Likert scale from 1 = *strongly disagree* to 7 = *strongly agree*. In this sample, Cronbach's alpha was 0.96, based on the imputed data.

#### 2.2.3 Diet

The participants were asked about their current diet. They could choose between the following forms of nutrition: omnivorous diet, vegetarian diet, vegan diet, flexitarian diet, paleo diet, and ketogenic diet. If none of the described diets applied to the participants, they could give their answer in an open format. The open responses were then either assigned to one of the described forms or new categories were created.

#### 2.2.4 Psychopathology

The participants were asked whether they had ever been diagnosed with a psychiatric disorder. If the answer to this question was *“yes”*, they were then asked about the specific diagnosis. Finally, the participants were asked to clarify whether the diagnosis was provided by their family doctor, psychiatrist, psychologist, psychotherapist, or another person.

In addition, scales on different psychological conditions that could be related to increased ON (e.g. EDE-Q) were included.

##### 2.2.4.1 Orthorexia nervosa

The Düsseldorf Orthorexia Scale (DOS) was used to record symptoms of orthorexic tendencies (Barthels, 2014). The scale consisted of a total of 10 items (e.g. *“If I eat something I consider unhealthy, I feel really bad”*), which were answered on a 4-point scale from 1 = *does not apply to me* to 4 = *applies to me*. A higher score indicated a higher level of orthorexic eating behavior and a cut-off value of 30 or above indicated the presence of ON. The cut-off was considered descriptively for sample characterization. Cronbach's alpha (MI) for the scale was 0.88.

##### 2.2.4.2 Eating disorder symptomatology

To assess eating disorder symptoms, the German version of the Eating Disorder Examination-Questionnaire (EDE-Q) 6.0 was used. The EDE-Q is the questionnaire version of the structured eating disorder interview, the Eating Disorder Examination (EDE; Fairburn et al., 2014, Fairburn et al., 1993, Hilbert and Tuschen-Caffier, 2016b), and is used to systematically record specific eating disorder psychopathology in adults and adolescents. The questionnaire consisted of a total of 28 items that were divided into four subscales: Restraint (e.g. “*Have you been deliberately trying to limit the amount of food you eat to influence your shape or weight [whether or not you have succeeded]?”*), Eating Concern (e.g. “*Has thinking about food, eating or calories made it very difficult to concentrate on things you are interested in [for example, working, following a conversation, or reading]?”*), Weight Concern (e.g. “*Have you had a strong desire to lose weight?”*) and Shape Concern (e.g. “*Have you felt fat?”*). Answers were given on a 7-point scale from 0 = *not at all* to 6 = *every day* or 0 = *not at all* to 6 = *markedly*. In this study, Cronbach's alpha (MI) was 0.82 for the subscale Restraint, 0.86 for Eating Concern, 0.84 for Weight Concern, and 0.92 for Shape Concern. Cronbach's alpha (MI) for the total score was 0.91.

##### 2.2.4.3 Hypochondria

The German version of the Whiteley Index was used to measure hypochondriacal attitudes and beliefs. The inventory consists of a total of 14 items that are answered on a dichotomous (yes/no) scale (Glöckner-Rist et al., 2007). A higher score indicates a higher probability of hypochondria. In this study, Cronbach's alpha was 0.83, based on the imputed data.

#### 2.2.5 Physical health

In line with psychopathology, the participants were also asked about their physical health. Firstly, participants were asked whether they had a chronic illness. If the answer to this question was *“yes”*, the participants were asked to specify the type of illness and whether it had been diagnosed by their family doctor, a specialist, or another person.

#### 2.2.6 Personality factors

##### 2.2.6.1 Negative emotionality

The German version of the Big Five Inventory 2 (BFI-2) (Danner et al., 2016) was used to assess personality factors that may be associated with higher DOS scores. The BFI-2 measures the Big Five personality traits Extraversion, Agreeableness, Conscientiousness, Negative Emotionality, and Openness. In this study, only the factor of Negative Emotionality with its three facets of Anxiety, Depression, and Emotional Volatility was assessed. Each facet included four items, e.g. *“I am often worried”* (Anxiety), *“I often feel depressed, without joy”* (Depression), and *“I can be moody and have fluctuating moods”* (Emotional Volatility), which were answered on a 5-point Likert scale from 1 = *strongly disagree* to 5 = *strongly agree*. In this sample, Cronbach's alpha (MI) was 0.78 for Anxiety, 0.86 for Depression, and 0.84 for Emotional Volatility. Cronbach's alpha for the total score was 0.91, based on the imputed data.

##### 2.2.6.2 Perfectionism

The German version of the Multidimensional Perfectionism Scale (Stöber, 1995) by Frost, Marten, Lahart and Rosenblate (Frost et al., 1990) was implemented to measure perfectionism, using the six dimensions of Concern over Mistakes (e.g. *“I rightly get upset when I make a mistake”)*, Doubts about Actions (e.g. *“Even with the simple everyday things I do, I usually have doubts”*), Parental Expectations (e.g. *“My parents set very high standards for me”*), Parental Criticism (e.g. “*As a child, I was punished if I didn't do things perfectly*”), Personal Standards (e.g. *“It is important for me to be fully competent in everything I do”*), and Organization (e.g. *“I am a tidy person”*) with a total of 35 items. The items were rated on a 5-point Likert scale from 1 = *strongly disagree* to 5 = *strongly agree*. Cronbach's alpha (MI) was 0.93 for Concern over Mistakes, 0.8 for Doubts about Actions, 0.91 for Parental Expectations, 0.87 for Parental Criticism, 0.85 for Personal Standards, and 0.89 for Organization. Cronbach's alpha for the total score was 0.81, based on the imputed data.

#### 2.2.7 Body acceptance

The Body Acceptance Scale of the Dresden Body Image Questionnaire (DKB-35) (Pöhlmann et al., 2013) was used to measure the extent to which the participants accepted their body as it was and were happy with it. The scale consisted of a total of eight items (e.g. *“I am satisfied with my appearance”*), which were answered on a 5-point Likert scale from 1 = *not at all* to 5 = *completely*. A higher value indicated greater acceptance of one's own body. In this study, Cronbach's alpha (MI) was 0.91.

#### 2.2.8 Self-esteem

To measure self-esteem, the German version of the Rosenberg Self-Esteem Scale (RSES) (Von Collani and Herzberg, 2003) was used. This scale measures global self-esteem with a total of 10 items (e.g. *“All in all, I am satisfied with myself”*), which were answered in this study on a 4-point Likert scale from 1 = *strongly disagree* to 4 = *strongly agree*. A higher value indicated higher self-esteem. In this sample, Cronbach's alpha was 0.92, based on the imputed data.

#### 2.2.9 Self-control

The self-control dimension of the Achievement Motivation Inventory (AMI) was used to assess motivational aspects. The AMI contains the most important dimensions that are addressed in various achievement motivation theories and mainly relates to aspects relevant to professional success. The self-control dimension consists of a total of 10 items (e.g. *“Before I start a new task, I always make a work plan first”*) and is based on the type of organization and execution of tasks. People with high scores are characterized by the fact that they are generally organized, do not postpone completion, and can easily concentrate on tasks (Schuler and Prochaska, 2001). These items were answered on a 7-point Likert scale from 1 = *strongly disagree* to 7 = *strongly agree*. Cronbach's alpha (MI) value was 0.73.

#### 2.2.10 Sociodemographic and anthropometric data

Lastly, demographic and anthropometric data including gender, age, residence (country), height, weight, education, and profession were collected.

### 2.3 Data analyses

All analyses were conducted with R (R Core Team, 2022). The open response fields were summarized and coded. For the variable *diet type*, open answers were analyzed retrospectively and the categories “gluten-free diet” and “pescetarian diet” were subsequently added. Information that did not fit into any of the specified categories was aggregated in the “no classification” category. The types of sports were also analyzed retrospectively and grouped into the following categories: acrobatics, endurance sports (including swimming or running), badminton, basketball, bouldering and climbing, cheerleading, crossrope, fitness (including aqua fitness or gym), soccer, golf, handball, hula hooping, martial arts (including boxing), bowling, strength and endurance training mixed (including jogging and strength training), strength training (including bodybuilding), athletics, horse riding, rope skipping, dancing (including ballet), tennis, table tennis, gymnastics, volleyball, water sports, yoga and Pilates, disc golf, ice skating, dog sports, motor sports, ultimate frisbee, winter sports (including skiing) and Zumba®. Lastly, participants were also able to provide information on their (current) psychiatric diagnosis in an open response field. For participants who provided several diagnoses, each diagnosis was analyzed individually. The diagnoses were categorized according to the current, established classification system (ICD-10).

The Multiple Imputation by Chained Equations (MICE) method was used to treat missing data. A threshold of 50% missing data was defined to ensure robust results (Junaid et al., 2025). Sociodemographic and grouping variables were not imputed to avoid biased results. Therefore, group differences were only calculated if the respective grouping variable was included in the original dataset. Results marked with MI are derived from data that has been imputed using multiple imputation (MI).

The mean values (*M*), standard deviations (*SD*) and absolute and relative frequencies have been presented in tabular form for the descriptive data. Confidence intervals (CI) were reported where appropriate to indicate the precision of the estimates. Prior to the inferential statistical analyses, the prerequisites for the tests were verified, while the normal distribution of the data was graphically checked using a histogram. Levene's test was used to test for homoscedasticity and independent t-tests were implemented to examine group differences in the severity of orthorexic tendencies. Similarly, we used classical one-way ANOVA and conducted pairwise comparisons with Holm's correction, pooling estimates across multiple imputation via Rubin's rules. When homogeneity of variances was violated, Welch's ANOVA with Games-Howell post-hoc tests was performed. Pearson correlation coefficient (*r*) was used to examine relationships between orthorexic tendencies and other metric variables. Partial correlations were computed to assess the associations between orthorexic tendencies and other metric variables while controlling for eating disorder symptomatology. The effect size was expressed as Hedges' *g* or ω^2^. An alpha level of 0.05 was used for all analyses, adjusted for false discovery rate (FDR) using Benjamini and Hochberg correction, as per Schnorr et al. (2024).

Hierarchical multiple linear regression was performed to examine the variables independently associated with orthorexic tendencies. The order of the blocks was based on the associated factors proposed by Donini et al. (2022), proceeding from distal control variables (e.g. sociodemographics like age) to proximal clinical factors. The first block comprised sociodemographic variables (e.g. age or gender), while the second included lifestyle factors (e.g. physical activity or diet). Psychological characteristics (e.g. perfectionism or self-esteem) were included in the third block. Finally, the fourth block comprised clinical variables, such as diagnosis of mental disorders, the EDE-Q and the Whiteley Index. Additionally, a sensitivity analysis was conducted with clinical variables entered in the first block to examine the incremental value of non-clinical predictors. The homoscedasticity assumption was tested using the Breusch-Pagan test. If violated, heteroscedasticity-consistent standard errors (HC3) were calculated and reported in the robustness analysis to avoid biased estimates of the standard errors. The models were estimated for all imputations and pooled according to Rubin's rules. Proportion of explained variance (*R*^2^) and change in explained variance (*ΔR*^2^) were reported to assess model quality, along with variance inflation factors (VIF) to control for multicollinearity.

## 3 Results

A total of 1842 datasets were collected. Of these, *n* = 311 datasets had to be removed as they did not meet the inclusion criteria: *n* = 204 only answered a maximum of one question, *n* = 2 did not meet the required minimum age of 18 years, *n* = 103 did not live in Germany, and *n* = 2 did not meet the required minimum age of 18 years and did not live in Germany. In addition, *n* = 482 participants were excluded from data analysis as they did not reach the required threshold for multiple imputation. In total, *n* = 1049 participants were included in data analyses with *n* = 985 complete datasets; this represented a total of *n* = 884 females (90.1 %), *n* = 91 males (9.3 %) and *n* = 6 other (0.6 %). The average age was *M* = 35.46 years (*SD* = 11.75) and the average BMI was *M* = 23.68 (*SD* = 5.01) kg/m^2^. Most participants reported a higher education entrance qualification (German Abitur; *n* = 190), a Bachelor's degree (*n* = 190), or a Master's degree (*n* = 144) as their highest level of education. *N* = 827 participants (84.3 %) were engaging in at least one type of sport: amongst these, *n* = 505 (60.8 %) also participated in a second type of sport, and *n* = 30 (3.6 %) specified that they were involved in competitive sports. The most frequently practiced type of sport was endurance training (27.4 %), followed by strength training (24.5 %), and fitness (11.9 %). On average, the participants engaging in sports trained for 3.92 days a week (*SD* = 1.56), with an average training duration of *M* = 68.02 (*SD* = 32.63) minutes. Most participants (39.3 %) described the average effort of their training over the previous 4 weeks as *exhausting*. Instagram was the most frequently used network with 97.2 % of the participants using this platform. Most participants (*n* = 814) used Instagram *several times a day,* with an average usage of *M* = 66.19 min (*SD* = 47.59 min) per day (Table 1).

**Table 1:** Overview of social media usage.

	Number of users (in %)	Frequency of usage	Duration of use per day (in minutes)	
			M	SD
Facebook	510 (52.0 %)	less than once a month	13.89	25.17
Instagram	954 (97.2 %)	several times a day	66.19	47.59
Threads	204 (20.8 %)	less than once a month	6.56	12.51
YouTube	909 (92.7 %)	one to three times a month	26.74	36.82
Pinterest	582 (59.4 %)	less than once a month	6.93	12.44
Snapchat	303 (30.9 %)	several times a day	10.72	14.98
TikTok	231 (23.6 %)	several times a day	38.44	56.48
X	138 (14.1 %)	less than once a month	8.48	17.83
Tumblr	82 (8.4 %)	less than once a month	2.62	8.31

*Notes*. *M* = mean, *SD* = standard deviation. The details regarding the average time of use per day only include the specified time of the people who used the network. Frequency of usage indicates the category that was selected by most participants.

The majority of participants followed an omnivorous (*n* = 335; 34.1 %), flexitarian (*n* = 297; 30.3 %), or vegetarian (*n* = 220; 22.4 %) diet. In total, *n* = 353 (36.0 %) participants reported having been diagnosed with a mental disorder at some point and *n* = 351 (35.8 %) with a chronic somatic disease. Out of these, the most frequently reported mental disorders were depressive disorder (*n* = 198; 56.4 %), anxiety disorder (*n* = 70; 19.9 %), and anorexia nervosa (*n* = 69; 19.7 %). In total, *n* = 120 (34.2 %) participants reported having some form of eating disorder or had suffered from one in the past. Table 2 details the descriptive characteristics of the examined scales; Table A.1 the correlations between the examined scales. The mean DOS score in the sample fell below the established cut-off for ON. In total, *n* = 127 (13.0 %) of participants scored at or above the DOS cut-off (≥30). Distributional diagnostics and visual inspection of histograms indicated no substantial deviations from normality for DOS scores.

**Table 2:** Descriptive characteristics of the key variables.

Scale	Scale range	*M*	*SD*	*n*
ON	10-40	21.28	6.41	1049
Eating disorder symptomatology	0-6	1.84	1.43	1049
Restraint	0-6	1.85	1.67	1049
Shape Concern	0-6	2.41	1.71	1049
Weight Concern	0-6	2.04	1.67	1049
Eating Concern	0-6	1.06	1.33	1049
Hypochondria	0-14	4.21	3.39	1049
Negative Emotionality	1-5	2.94	0.77	1049
Anxiety	1-5	3.27	0.82	1049
Depression	1-5	2.69	0.94	1049
Emotional Volatility	1-5	2.85	0.89	1049
Perfectionism	29-145	78.84	22.77	1049
Concern over Mistakes	9-45	24.39	8.98	1049
Personal Standards	7-35	22.22	6.19	1049
Parental Expectations	5-25	11.8	5.38	1049
Parental Criticism	4-20	9.58	4.46	1049
Doubts about Actions	4-20	10.85	3.96	1049
Organization	6-30	23.92	4.48	1049
Body Acceptance	1-5	3.14	0.88	1049
Self-esteem	10-40	30.94	7.08	1049
Self-control	10-70	43.12	9.88	1049
Engagement in health and fitness content on social media	1-7	5.2	1.63	1049

*Notes*. *M* = mean, *SD* = standard deviation, *n* = number of participants. All depicted values are based on imputed data.

### 3.1 Sociodemographic and anthropometric data and orthorexic tendencies

There was no significant correlation between orthorexic tendencies and age, *r*_MI_ = −0.04, *p* > .05, whereas a significant small negative correlation of *r*_MI_ = −0.11, *p =* .002, was found between BMI and orthorexic tendencies. No significant differences in DOS scores were observed between women and men, *t*_MI_(812) = −2.04, *p* > .05, 95 % CI [−3.08, −0.06], *g* = −0.24, and educational qualifications, *F*_MI_(7, 901) = 1.49, *p* > .05, ω^2^ = .004.^2^

### 3.2 Physical activity and orthorexic tendencies

Participants who engaged in sports had significantly higher orthorexic tendencies than those who did not, *t*_MI_(977) = 3.36, *p* = .01, 95 % CI [0.79, 3.01], *g* = 0.29. However, no significant differences were found between individuals who practiced competitive sports compared to those who did not, *t*_MI_(826) = 2.01, *p > .05*, 95 % CI [0.06, 4.83], *g* = 0.37. Also, no differences in DOS scores between participants who practiced only one and those who practiced several types of sport, *t*_MI_(821) = −0.55, *p* > .05, 95 % CI [−1.18, 0.66], *g* = −0.04, were observed. Accordingly, the one-way ANOVA revealed no significant differences in the DOS scores between the different types of sport, *F*_*MI*_(25, 800) = 0.751, *p* > .05, ω^2^ < 0.0001.

There was a small significant correlation of *r*_MI_ = 0.2, *p* < .001, between orthorexic tendencies and days of training per week. Significant group differences in DOS scores between different training days, *F*_MI_(6, 231.15) = 9.58, *p* < .001, ω^2^ = 0.18, were observed, with results of the post hoc analysis shown in Table 3. Contrarily, no significant correlations between orthorexic tendencies and the average training time (in minutes) per training session, *r*_MI_ = −0.001, *p* > .05, or training hours per week, *r*_MI_ = 0.03, *p* > .05, were observed. Regarding effort, however, there was a small, significant correlation of *r*_MI_ = 0.09, *p* = .04, with orthorexic tendencies. ANOVA showed significant group differences in DOS scores between different effort levels, *F*_MI_(8, 817) = 2.33, *p* = .035, ω^2^ = 0.01, with pairwise comparisons only revealing significant group differences between individuals who described their training as *extremely low exhausting* and those who described it as *extremely exhausting* (*ΔM* = 10.6, *p* = .038).

**Table 3:** Mean differences in DOS scores between groups with different training days per week.

Days of training	1 per week	2 per week	3 per week	4 per week	5 per week	6 per week
**2 per week**	3.34∗∗∗					
**3 per week**	3.72∗∗∗	0.38				
**4 per week**	3.5∗∗∗	0.16	−0.22			
**5 per week**	4.3∗∗∗	0.95	0.57	0.79		
**6 per week**	7.05∗∗∗	3.71∗∗∗	3.33∗∗∗	3.55∗∗∗	2.76∗∗∗	
**7 per week**	8.06∗∗∗	4.72∗∗∗	4.34∗∗∗	4.56∗∗∗	3.77∗∗∗	1.01

*Notes*. Mean differences (Games-Howell) of DOS scores (MI) are shown as absolute values; ∗∗∗ indicates *p* < .001.

### 3.3 Social media and orthorexic tendencies

There was a moderate significant correlation of *r*_MI_ = 0.35, *p* < .001, between orthorexic tendencies and the engagement with health and fitness content on social media. Contrarily, there was no significant difference in the DOS scores between people who used Facebook, *t*_MI_(976) = 0.88, *p* > .05, 95 % CI [−0.45, 1.18], *g* = −0.06, Instagram, *t*_MI_(977) = −1.83, *p* > .05, 95 % CI [−4.79, 0.17], *g* = 0.36, YouTube, *t*_MI_(977) = −0.44, *p* > .05, 95 % CI [−1.91, 1.21], *g* = 0.05, Pinterest, *t*_MI_(975) = −0.39, *p* > .05, 95 % CI [−1.0, 0.66], *g* = 0.03, Snapchat, *t*_MI_(974) = 1.77, *p* > .05, 95 % CI [−0.09, 1.67], *g* = −0.12, TikTok, *t*_MI_(972) = 0.06, *p* > .05, 95 % CI [−0.93, 0.99], *g* = 0.0, X, *t*_MI_(970) = 0.97, *p* > .05, *g* = −0.09, 95 % CI [−0.59, 1.75], Tumblr, *t*_MI_(962) = 0.51, *p* > .05, 95 % CI [−1.09, 1.85], *g* = −0.06, or Threads, *t*_MI_(972) = −0.33, *p* > .05, 95 % CI [−1.17, 0.83], *g* = 0.03, and those who did not.

There were also no significant differences in the DOS scores between the social network used most frequently, *F*_*MI*_(8, 855) = 1.38, *p* > .05, ω^2^ = 0.004. However, there was a small, significant correlation between orthorexic tendencies and time (in minutes) spent on Instagram, *r*_MI_ = 0.07, *p* = .038, and Pinterest, *r*_MI_ = 0.07, *p* = .029, on an average day. No significant correlation was shown between orthorexic tendencies and the time spent on Facebook, *r*_MI_ = 0.06, *p* > .05, Threads, *r*_MI_ = 0.01, *p* > .05, YouTube, *r*_MI_ = −0.02, *p* > .05, Snapchat, *r*_MI_ = −0.02, *p* > .05, TikTok, *r*_MI_ = 0.086 *p* > .05, X, *r*_MI_ = −0.04, *p* > .05, or Tumblr, *r*_MI_ = 0.03, *p* > .05.

### 3.4 Nutrition and orthorexic tendencies

Significant group differences of DOS scores between the types of nutrition, *F*_MI_(7.00, 26.68) = 4.28, *p* < .001, ω^2^ = 0.4, were observed. Post hoc analyses showed significant differences between flexitarian (*M* = 20.54, *SD* = 6.07) and vegan (*M* = 23.84, *SD* = 7.5) diets (*ΔM* = 3.3, *p* < .001), flexitarian (*M* = 20.54, *SD* = 6.07) and vegetarian (*M* = 22.76, *SD* = 6.55) diets (*ΔM* = 2.22, *p* < .001), omnivore (*M* = 20.34, *SD* = 6.14) and vegan (*M* = 23.84, *SD* = 7.5) diets (*ΔM* = 3.5, *p* < .001), and omnivore (*M* = 20.34, *SD* = 6.14) and vegetarian (*M* = 22.76, *SD* = 6.55) diets (*ΔM* = 2.42, *p* < .001).

### 3.5 Psychopathology and orthorexic tendencies

Individuals who have or who had had a mental disorder showed significantly higher DOS scores compared to those who had never had a mental disorder, *t*_MI_(977) = 8.23, *p* < .001, 95 % CI [2.62, 4.26], *g* = 0.55. Furthermore, people with a current or past diagnosis of an eating disorder revealed significantly higher DOS scores compared to those who did not, *t*_MI_(1006) = 11.53, *p* < .001, 95 % CI [5.63, 7.95], *g* = 1.12. When particular diagnoses were considered, individuals with a current or past diagnosis of anorexia nervosa (F50.0) had higher orthorexic tendencies than people who have never been diagnosed with anorexia nervosa, *t*_MI_(1032) = 8.02, *p* < .001, 95 % CI [4.71, 7.76], *g* = 1.00. Higher DOS scores were detected for individuals who presented with, or who had, a previous diagnosis of depression (F32, F33) compared to those without a diagnosis, *t*_MI_(957) = 4.68, *p* < .001, 95 % CI [1.37, 3.36], *g* = 0.37. In addition, individuals with a current or past diagnosis of anxiety disorder (F40, F41) had higher DOS scores than those who had never been diagnosed, *t*_MI_(1032) = 2.63, *p* = .036, 95 % CI [0.53, 3.65], *g* = 0.33. Ultimately, t-tests revealed no differences in the DOS scores between individuals with a current or past diagnosis of OCD and those without a diagnosis, *t*_MI_(978) = 1.81, *p* > .05, 95 % CI [−0.26, 6.24], *g* = 0.47.

There was a strong, significant correlation of *r*_MI_ = 0.62, *p* < .001, between the EDE-Q score and orthorexic tendencies, and a moderate correlation of *r*_MI_ = 0.34, *p* < .001, between the Whiteley Index and orthorexic tendencies.

### 3.6 Chronic disease and orthorexic tendencies

The t-tests revealed no significant differences in the DOS scores between people who suffered from a chronic disease and those who did not, *t*_MI_(976) = 1.85, *p* > .05, 95 % CI [−0.05, 1.64], *g* = 0.12.

### 3.7 Personality factors and orthorexic tendencies

There was a moderate, significant correlation of *r*_MI_ = 0.34, *p* < .001*,* between the DOS scores and the overall score for Negative Emotionality. Furthermore, the more specific facet traits, i.e. Anxiety, *r*_MI_ = 0.26, *p* < .001*,* Depression, *r*_MI_ = 0.38 *p* < .001*,* and Emotional Volatility, *r*_MI_ = 0.23, *p* < .001*,* showed significant correlations with orthorexic tendencies.

### 3.8 Perfectionism and orthorexic tendencies

There was a moderate, significant correlation of *r*_MI_ = 0.43, *p* < .001, between the DOS scores and overall perfectionism score. Similarly, all subscales showed significant correlations with orthorexic tendencies: Personal Standards, *r*_MI_ = 0.37, *p* < .001, Organization, *r*_MI_ = 0.2, *p* < .001, Concern over Mistakes, *r*_MI_ = 0.41, *p* < .001, Doubts about Actions, *r*_MI_ = 0.27, *p* < .001, Parental Expectations, *r*_MI_ = 0.24, *p* < .001, and Parental Criticism, *r*_MI_ = 0.3, *p* < .001.

### 3.9 Self-concept and orthorexic tendencies

The Body Acceptance Scale was significantly negatively correlated with orthorexic tendencies, *r*_MI_ = −0.41, *p* < .001. Equally, self-esteem, *r*_MI_ = −0.41, *p* < .001, and self-control, *r*_MI_ = 0.11, *p* = .002, were significantly correlated with orthorexic tendencies. The correlations remained significant even after controlling for eating disorder symptoms (Table A.2).

### 3.10 Hierarchical multiple regression

The predictors were introduced in blocks in a hierarchical regression model (Table 4). Variance inflation factors (VIF) ranged from 1.02 to 1.86, indicating no problematic multicollinearity among predictors. Sociodemographic and anthropometric variables (age, gender and BMI) explained 1.5 % of the variance (*R*^*2*^ = 0.015). Adding lifestyle factors in the second block (physical activity, training days per week, diet and engagement with health and fitness content on social media) increased the explained variance significantly by *ΔR*^*2*^ = 0.19, *F*_MI_(10, 939) = 22.18, *p < .001*, so that a total of 20.0 % of the variance was explained (*R*^*2*^ = 0.20). In the third block, psychological variables (negative emotionality, perfectionism, body acceptance, self-esteem and self-control) were added. The proportion of explained variance increased significantly by *ΔR*^*2*^ = 0.22, *F*_MI_(5, 934) = 70.67, *p < .001,* explaining a total of 42.0 % of the variance (*R*^*2*^ = 0.42). Finally, the inclusion of clinical variables (diagnosis of eating disorder, diagnosis of anorexia nervosa, diagnosis of depression, diagnosis of anxiety disorder, diagnosis of OCD, eating disorder symptoms according to EDE-Q, as well as hypochondria) in the fourth block increased the explained variance by *ΔR*^*2*^ = 0.16, *F*_MI_(7, 927) = 49.29, *p < .001.* Consequently, the overall model explained 57.7 % of the variance (*R*^*2*^ = 0.58). The results of the final regression model are illustrated in Figure A.1.

**Table 4:** Hierarchical Regression Results for orthorexic tendencies.

Variable	B	95% CI for B		SE B	β	R^2^	ΔR^2^
		LL	UL				
Step 1						0.01	
Constant	23.44∗∗∗	20.90	25.98	1.30			
Age	−0.01	−0.05	0.02	0.02	−0.02		
Gender	1.29	−0.09	2.66	0.70	-		
BMI	−0.12∗∗	−0.21	−0.04	0.04	−0.10∗∗		

Step 2						0.20	0.19∗∗∗
Constant	12.04∗∗∗	9.21	14.88	1.45			
Age	−0.03	−0.06	0.01	0.02	−0.05		
Gender	0.18	−1.15	1.51	0.68	-		
BMI	−0.03	−0.12	0.05	0.04	−0.03		
Physical activity	0.60	−0.38	1.57	0.50	-		
Training days per week	0.61∗∗∗	0.34	0.88	0.14	0.15∗∗∗		
Diet							
Flexitarian diet	0.28	−0.62	1.17	0.46	-		
Vegetarian diet	2.07∗∗∗	1.06	3.08	0.52	-		
Vegan diet	3.04∗∗	1.15	4.92	0.96	-		
Pescetarian diet	−0.48	−4.01	3.06	1.80	-		
Ketogenic diet	6.23∗	1.0	11.47	2.67	-		
Paleo diet	−0.23	−2.63	2.17	1.22	-		
Gluten-free diet	0.94	−2.79	4.67	1.90	-		
Content	1.37∗∗∗	1.12	1.62	0.13	0.35∗∗∗		

Step 3						0.42	0.22∗∗∗
Constant	15.26∗∗∗	9.83	20.69	2.77			
Age	0.03∗	0.0	0.06	0.02	0.06∗		
Gender	−0.93	−2.10	0.24	0.60	-		
BMI	−0.10∗	−0.18	−0.02	0.04	−0.08∗		
Physical activity	1.76∗∗∗	0.85	2.67	0.46	-		
Training days per week	0.44∗∗∗	0.21	0.66	0.12	0.11∗∗∗		
Diet							
Flexitarian diet	0.32	−0.45	1.10	0.39	-		
Vegetarian diet	1.48∗∗∗	0.64	2.33	0.43	-		
Vegan diet	2.25∗∗	0.70	3.79	0.79	-		
Pescetarian diet	0.41	−2.93	3.74	1.70	-		
Ketogenic diet	5.91∗	0.78	11.04	2.62	-		
Paleo diet	−0.82	−4.40	2.76	1.83	-		
Gluten-free diet	−0.30	−4.43	3.82	2.11	-		
Content	1.12∗∗∗	0.89	1.35	0.12	0.28∗∗∗		
Negative Emotionality	0.46	−0.21	1.12	0.34	0.06		
Perfectionism	0.05∗∗∗	0.03	0.07	0.01	0.16∗∗∗		
Self-esteem	−0.12∗∗	−0.20	−0.04	0.04	−0.13∗∗		
Self-control	0.06∗∗∗	0.03	0.10	0.02	0.10∗∗∗		
Body Acceptance	−1.78∗∗∗	−2.30	−1.25	0.27	−0.24∗∗∗		

Step 4						0.58	0.16∗∗∗
Constant	4.64	−0.35	9.63	2.55			
Age	0.03∗	0.00	0.06	0.01	0.05∗		
Gender	1.54∗∗	0.47	2.62	0.55	-		
BMI	−0.18∗∗∗	−0.25	−0.11	0.03	−0.14∗∗∗		
Physical activity	1.10∗∗	0.31	1.89	0.40	-		
Training days per week	0.22∗	0.01	0.43	0.11	0.05∗		
Diet							
Flexitarian diet	0.24	−0.44	0.92	0.35	-		
Vegetarian diet	1.07∗∗	0.36	1.79	0.37	-		
Vegan diet	2.12∗∗	0.75	3.48	0.69	-		
Pescetarian diet	0.62	−2.19	3.44	1.44	-		
Ketogenic diet	3.59	−0.88	8.07	2.28	-		
Paleo diet	1.63	−3.19	6.44	2.46	-		
Gluten-free diet	0.34	−3.26	3.94	1.84	-		
Content	0.83∗∗∗	0.62	1.04	0.11	0.21∗∗∗		
Negative Emotionality	0.17	−0.42	0.76	0.30	0.02		
Perfectionism	0.02∗	0.00	0.04	0.01	0.07∗		
Self-esteem	−0.06	−0.13	0.01	0.04	−0.06		
Self-control	0.07∗∗∗	0.04	0.10	0.02	0.11∗∗∗		
Body Acceptance	1.10∗∗∗	0.58	1.61	0.26	0.15∗∗∗		
Eating disorder	2.23∗∗	0.76	3.69	0.75	-		
Anorexia nervosa	−1.43	−3.20	0.34	0.90	-		
OCD	0.55	−2.29	3.38	1.45	-		
Anxiety disorder	−0.11	−1.45	1.23	0.68	-		
Depression	0.10	−0.71	0.91	0.41	-		
EDE-Q	2.66∗∗∗	2.35	2.98	0.16	0.60∗∗∗		
Hypochondria	0.25∗∗∗	0.14	0.36	0.06	0.13∗∗∗		

*Notes*. B = unstandardized regression coefficient, CI = confidence interval, LL = lower limit, UL = upper limit, SE B = Standard error, β = standardized regression coefficient; Content: engagement with health and fitness content on social media; ∗*p* < .05, ∗∗*p* < .01, ∗∗∗*p* < .001. All depicted values are based on imputed data.

To verify the robustness of the results, a sensitivity analysis was performed in which the clinical variables were first included in the model. This alone explained 44.3 % of the variance (*R*^*2*^ = 0.44). The subsequent inclusion of sociodemographic variables led to a significant, albeit small, increase in explained variance, *ΔR*^*2*^ = 0.04, *F*_MI_(3, 964) = 28.05, *p < .001.* Lifestyle factors increased the explained variance by *ΔR*^*2*^ = 0.07, *F*_MI_(10, 932) = 15.26, *p < .001,* while personality traits contributed a modest but significant amount, *ΔR*^*2*^ = 0.02, *F*_MI_(5, 927) = 9.92, *p < .001.* Overall, the final model explained 57.7 % of the variance (*R*^*2*^ = 0.58), thus confirming the main results of the analysis.

## 4 Discussion

Following the framework for valid ON diagnosis proposed by Donini et al. (2022), this study with over 1000 participants was the first to examine, systematically, the cross-sectional associations between orthorexic tendencies and the factors highlighted by the ON task force, thus providing a broad overview of how these variables were related to orthorexic tendencies. The main results of this study were: (1) In the present sample, individuals with a current or past diagnosis of a mental disorder, especially an eating disorder, revealed significantly higher orthorexic tendencies than individuals without a mental disorder history, (2) significant correlations exist between DOS scores and the EDE-Q score, perfectionism, body acceptance, engagement with health and fitness content, BMI, and training days, and (3) significantly higher orthorexic tendencies in physically active participants are independent of the type of sport.

Hierarchical regression analysis also revealed the incremental explanatory value of different variable groups. Demographic and anthropometric variables alone explained only 1.5 % of the variance in DOS scores. The incorporation of lifestyle factors resulted in a substantial augmentation of the explained variance by 18.9 %, and personality traits by an additional 22.0 %. Finally, the additional 15.7 % of the variance was explained by the addition of clinical variables, so that 57.7 % of the variance was explained by the overall model. Sensitivity analysis confirmed these results, showing that clinical variables alone explained 44.3 % of the variance. In the present sample, these results indicate that orthorexic tendencies were most strongly embedded in eating disorder-related psychopathology. Although psychological and behavioral factors also contributed to the overall pattern of associations, clinical variables accounted for the largest proportion of explained variance, indicating the particular importance of eating disorder symptomatology and broader psychopathology. The importance of psychopathology is particularly emphasized in our sample when we consider individuals with a current or past mental disorder, who exhibited significantly higher DOS scores (*p* < .001). So far, ON has not been viewed as a distinct disorder in the current diagnostic systems (DSM-5 or ICD-10) and has not been included in ICD-11. Previous research has debated overlaps of ON with both eating disorders and OCD (Cosh et al., 2023); in our study, the strongest effect (*g*_MI_ = 1.12, *p* < .001) emerged for individuals with a current or past eating disorder, who exhibited significantly higher DOS scores than those without an eating disorder history. The relationship remained consistent when controlling for other variables (*p* = .002). Similarly, there was a strong correlation between DOS scores and eating disorder symptoms (EDE-Q; *r*_MI_ = 0.62, *p* < .001). This was also reflected in the multivariate model, in which eating disorder symptoms showed the strongest independent association with orthorexic tendencies (*p* < .001). These findings suggest that DOS scores were closely associated with eating disorder symptomatology and that orthorexic tendencies in the present sample were related to broader psychopathology, with the strongest overlap observed for eating disorder symptomatology. The strong association between DOS and EDE-Q scores, the markedly elevated DOS scores among individuals with a current or past eating disorder, the prominent role of eating disorder symptomatology in the multivariate analyses, and the sensitivity analysis showing that clinical variables alone explained a substantial proportion of variance in DOS scores all point to a substantial construct overlap. This pattern raises concerns about the discriminant validity of orthorexic tendencies and argues against a strong separation of orthorexic tendencies from eating disorder psychopathology. At the same time, this overlap does not necessarily preclude some degree of specificity, particularly if orthorexic tendencies are conceptualized as an eating disorder-related phenotype rather than as a clearly independent construct. Since the EDE-Q is designed to assess eating disorder psychopathology broadly (Hilbert and Tuschen-Caffier, 2016) and has shown good ability to distinguish individuals with eating disorders from nonclinical controls (Berg et al., 2012), a substantial association with DOS scores would be expected if orthorexic tendencies are situated within the eating disorder spectrum. Importantly, several associations with orthorexic tendencies remained statistically significant after controlling for eating disorder symptoms in the partial correlations. In the present sample, this may suggest that the associations between orthorexic tendencies and several correlates were not fully explained by broader eating disorder symptoms alone and that orthorexic tendencies may capture additional variance beyond what is assessed by the EDE-Q. This residual variance should not be interpreted as evidence for a construct that is clearly independent from eating disorder psychopathology. Rather, it may indicate that orthorexic tendencies represent a more specific health-, purity-, and quality-oriented manifestation within the broader eating disorder spectrum, characterized by a predominant focus on perceived healthiness, food quality, bodily optimization, and self-regulatory control (Barthels and Pietrowsky, 2024; Cena et al., 2019; Donini et al., 2022; Horovitz and Argyrides, 2023). Although the present cross-sectional design does not allow conclusions as to whether orthorexic tendencies constitute an independent diagnostic entity, a subtype, or a transdiagnostic phenotype, the observed pattern of findings is compatible with the interpretation that orthorexic tendencies, in this health-focused and interest-driven sample, may be better understood as an eating disorder-related phenotype within the broader context of eating disorder psychopathology rather than as a construct clearly independent from it. Future research, especially longitudinal and clinical studies using diagnostic interviews and analytic approaches explicitly designed to examine discriminant and incremental validity, is needed to determine the diagnostic status of orthorexic tendencies within the broader eating disorder spectrum and to clarify whether they are better understood as a specific diagnostic entity or as a manifestation of broader eating disorder psychopathology.

In this sample no significant differences in DOS scores were found between individuals with a current or past diagnosis of OCD in either the bivariate or multivariate analyses. These results are in line with those of a previous study by Hessler-Kaufmann and colleagues (Hessler-Kaufmann et al., 2021), who showed that the prevalence of ON was highest in patients with eating disorders. Moreover, the study by Barthels et al. (2017) revealed that the prevalence of ON is significantly higher in people with eating disorders while it remains within the norm for people with OCD; similarly, numerous studies have shown this relationship between orthorexic symptoms and eating disorder symptoms (Bartel et al., 2020; Walker-Swanton et al., 2020; Zohar et al., 2023). Regarding OCD, however, the evidence is not clear; while some studies have exposed a significant positive correlation between orthorexic symptoms and OCD symptoms (e.g. Walker-Swanton et al., 2020), other studies have not (e.g. Dolapoglu et al., 2023). Taken together, the present findings support the view that orthorexic tendencies are more closely aligned with eating disorder symptomatology than with OCD.

Regarding eating disorder psychopathology, orthorexic eating behavior is often discussed in relation to anorexia nervosa. In our study, participants with anorexia nervosa (currently or in the past) had significantly higher DOS scores than those without the diagnosis of anorexia nervosa (*g*_MI_ = 1.00, *p* < .001). However, this association was no longer significant when other variables were controlled for in the multivariate model. In contrast, orthorexic tendencies remained significantly associated with eating disorder symptoms, suggesting that the bivariate association with anorexia nervosa may largely reflect shared variance with broader eating disorder psychopathology.

However, orthorexic tendencies appear to be associated with similar factors as anorexia nervosa such as BMI, perfectionism, body acceptance, and self-esteem. In our study, the bivariate analysis revealed significant correlations between DOS scores and BMI, *r*_MI_ = −0.11, *p* = .002, body acceptance, *r*_MI_ = −0.41, *p* < .001, self-esteem, *r*_MI_ = −0.41, *p* < .001, and perfectionism total score, *r*_MI_ = 0.43, *p* < .001, including all subscales (*p* < .001). In the multivariate regression model, BMI (*p* < .001), body acceptance (*p* < .001) and perfectionism (*p* = .019) remained significant as independent predictors. Notably, body acceptance showed a positive regression coefficient in the hierarchical regression model, although the bivariate associations suggested the opposite direction. More specifically, body acceptance was strongly negatively correlated with both orthorexic tendencies and eating disorder symptoms, whereas eating disorder symptoms were strongly positively associated with orthorexic tendencies. This pattern is consistent with a suppression effect, indicating that the association between body acceptance and orthorexic tendencies changed after accounting for variance shared with eating disorder psychopathology. Thus, the positive coefficient should not be interpreted as evidence that higher body acceptance is generally associated with higher orthorexic tendencies. Once eating disorder symptoms were controlled for, body acceptance appeared to capture variance not shared with eating disorder symptoms. One possible interpretation is that, after accounting for variance shared with eating disorder psychopathology, body acceptance may reflect a more positive body-related self-perception that is less closely tied to general eating disorder psychopathology and more closely related to attitudes such as perceived health, self-regulation, or bodily control. In this context, rigid “healthy” eating may be experienced as a way of caring for, regulating, or optimizing the body, which may help explain why this remaining component was positively associated with orthorexic tendencies.

Unlike body acceptance, self-esteem did not show a suppressor pattern in the multivariate model but was no longer significantly associated with orthorexic tendencies. Given that self-esteem was negatively associated with eating disorder symptoms, whereas eating disorder symptoms were positively associated with orthorexic tendencies, the negative bivariate association between self-esteem and orthorexic tendencies may largely reflect their shared association with broader eating disorder psychopathology. This pattern suggests that self-esteem may be a more general correlate of eating disorder psychopathology than a specific correlate of orthorexic tendencies.

Perfectionism is associated with a strong desire to control (Andrade et al., 2025) and may therefore be related to rigid dietary behaviors and high self-criticism. From a hypothesis-generating, neuroscience-informed perspective, this pattern may also be considered in relation to broader neurocognitive processes that have been discussed in eating disorder psychopathology. In particular, perfectionism has been linked to reduced cognitive flexibility (Buzzichelli et al., 2018; Miles et al., 2022), and the rigid adherence to “healthy” eating rules, a central feature of orthorexic eating behavior, may therefore be interpreted as potentially consistent with inflexible and overly controlled self-regulation. More broadly, this pattern may be compatible with a perfectionism-related style of rigid self-regulation and difficulty relaxing self-imposed dietary standards. Importantly, the present study did not assess neurocognitive performance or neural mechanisms directly. Thus, the considerations should be understood as theoretical interpretations that may help generate hypotheses for future research, rather than as mechanistic conclusions derived from the present data. Future studies using neurocognitive tasks, neuroimaging, or experimental designs are needed to examine whether the theoretical considerations are empirically relevant to orthorexic tendencies and to clarify similarities to and differences from related forms of psychopathology.

While our results regarding the association between orthorexic tendencies and perfectionism are in line with the current state of research (Barlow et al., 2024; Novara et al., 2023; Pratt et al., 2022), existing studies indicate a mixed pattern for the other factors: some studies have shown a correlation between higher orthorexic tendencies and low self-esteem (Bóna et al., 2021; Raynal et al., 2022), however, other studies reveal mixed results (Oberle et al., 2017; Sfeir et al., 2022). A similar picture emerges when considering body image dissatisfaction with some studies showing a relationship with orthorexic eating behavior (Barthels et al., 2021; Hessler-Kaufmann et al., 2021), whereas other studies do not (He et al., 2021; Waterman et al., 2022). Recent research regarding BMI also shows mixed results (Oberle et al., 2017; Strahler et al., 2018; Waterman et al., 2022), however our results support the finding of a negative, albeit weak, correlation.

The results of the present study suggest that orthorexic tendencies appear to differ from anorexia nervosa in some physical (BMI) and motivational characteristics. A low BMI is one of the diagnostic criteria for anorexia nervosa (Dilling and Freyberger, 2019). Contrarily, low BMI is not always present in individuals with orthorexic tendencies (Turner and Lefevre, 2017); this was also suggested by the small negative correlation revealed in this study. While the desire to lose weight and a negative body image, or the perception of being too weighty are motives that both underlie and are diagnostic criteria for anorexia nervosa (Dilling and Freyberger, 2019), ON is characterized primarily by an obsessive preoccupation with a supposedly healthy diet and does not necessarily translate to body mass loss or a distorted body image. Although anorexia nervosa and orthorexic tendencies are often discussed together in the literature, and appear to share certain features while also differing in important respects, the present findings do not permit conclusions as to whether ON should be conceptualized as a subtype of anorexia nervosa, a dimensional variant within the eating disorder spectrum, or a partially distinct phenomenon.

The ideal image of a “healthy” body is especially promoted on social media. Unsurprisingly, associations between DOS scores and engagement with health and fitness content on social media were observed in both bivariate (*r*_MI_ = 0.35, *p* < .001) and multivariate (*p* < .001) analyses. Our findings are in line with a recent study by Scheiber et al. (2023) which demonstrated a significant correlation between orthorexic tendencies and involvement in health and fitness accounts on social media. Consistent with this study, Levin et al. (2023) similarly revealed a relationship between ON scores and the amount of time spent daily on consuming content related to healthy eating on social media. There are numerous profiles on social media disseminating advice on fitness, nutrition and general health (Segado Fernández et al., 2025), often promoting rapid and effortless transformations towards a “healthy” body or diet. Such dichotomous portrayal of food and body image as either “healthy” or “unhealthy” may be associated with psychological pressure and the internalization of unrealistic health ideals, which in turn could be associated with stronger orthorexic tendencies, particularly in individuals already highly engaged with health-related content. Social media platforms use complex algorithms to prominently feature personalized content that individual users have previously engaged with (Griffiths et al., 2024). From a hypothesis-generating perspective, repeated exposure to health-, fitness-, and body-related content on social media may be associated with greater subjective salience of ideas related to “healthy” eating, health-related bodily optimization, or “proper” nutrition. Repeated engagement with such content may also be associated with the consolidation of underlying beliefs and the internalization of health- and body-related ideals, which could, in theory, be relevant to problematic eating-related cognitions and behaviors (Roorda and Cassin, 2025; Scheiber et al., 2023; Turner and Lefevre, 2017). However, because the present study relied on self-report measures and did not assess attentional, reward-related, or other neurocognitive processes directly, these interpretations should be regarded as theoretical considerations for future research rather than evidence of specific mechanisms.

Another factor that may be correlated with disordered eating behavior is participation in sports that promote a thin body ideal (Thiemann et al., 2015). In this study, however, no significant differences were observed across the various types of sports. The literature in this regard is mixed: some studies reporting increased orthorexic tendencies in yoga immersion (Domingues and Carmo, 2021) and aerobic exercise or strength training (Oberle et al., 2018), while other studies have found no specific association between orthorexic tendencies and the different types of sport (Bóna et al., 2019; Mavrandrea and Gonidakis, 2023). Individuals engaging in body-conscious sports may experience a greater (perceived) need to control their diet in order to achieve athletic goals, which could be associated with a more obsessive attitude towards diet and sports and, in turn, with stronger orthorexic tendencies. Another aspect that may be relevant to the observed association between sports and orthorexic tendencies, especially in recreationally trained individuals, is that sports are often closely linked to the idea of “health”. In some cases, this may be associated with an excessive focus on sports to optimize one's health and, in some cases, with a stronger preoccupation with health-related behaviors. For a more comprehensive understanding of the relationship between orthorexic eating behavior and sports it is necessary to consider the underlying motives of those affected.

Although no correlation was exposed for training hours per week, in the present sample, individuals who exercised regularly had higher levels of orthorexic tendencies than those who did not (*g*_MI_ = 0.29, *p* = .01), even after controlling for other variables (*p* = .006). In addition, correlations between DOS scores and the number of training sessions per week, *r*_MI_ = 0.2, *p* < .001, were observed, which was also evident in the multivariate model (*p* = .04). Recent studies indicate that higher levels of orthorexic tendencies may be linked to an increased participation in physical activity (Almeida et al., 2018; Martinovic et al., 2022b; Piko et al., 2024). Similar to our findings, Bóna et al. (2019) reported a significant correlation between orthorexic tendencies and training frequency per week. This association may be interpreted in the context of a broader health-oriented and control-related style of self-regulation, in which exercise and eating routines become closely intertwined. In some individuals, this may be associated with a more rigid form of self-regulation characterized by adherence to self-imposed health-related standards. However, methodological inconsistencies regarding mechano-biological descriptors of exercise, for example, those suggested by Toigo and Boutellier (2006), have resulted in the hindrance of in-depth analysis; these should be included in future cross-sectional studies that include physical activity-associated factors.

## 5 Limitations

Although the present study provides valuable insights into the relationship between orthorexic tendencies and potential associated factors, it has some limitations. The generalizability of the findings is limited by the specific characteristics of the sample. Recruitment was primarily carried out via social networks, particularly profiles that post health- and fitness-related content and this may have introduced a bias regarding the perceived importance of health. Individuals with a particular interest in diet and exercise or those who identify with orthorexic tendencies may have been over-represented. In addition, the sample was predominantly female and included a high proportion of participants with a self-reported history of mental disorders, particularly eating disorders. Taken together, these characteristics indicate that the sample was not representative of the general population but rather may reflect a self-selected subgroup with elevated health focus and psychological vulnerability. Collectively, the present sample may be more appropriately characterized as a self-selected, health-focused, psychologically vulnerable, and potentially higher-risk or interest-driven sample rather than a general population sample.

Furthermore, some subgroup analyses were based on relatively small subgroup sizes. In particular, the subgroup of competitive athletes was small (*n* = 30), and some diet-specific groups (e.g., ketogenic or paleo) were also small, limiting the statistical stability and robustness of these findings. Accordingly, these findings should be interpreted with caution, and the overall results should not be generalized to the general population. In addition, although the study provides a broad overview of potential associations and already takes a large number of possible confounding variables into account, not all relevant variables (e.g. perceived control, general tendencies towards eating disorders or social comparison) were included. Although some findings are interpreted from a neuroscience-informed perspective, the present study relied exclusively on self-report psychometric measures. Accordingly, these interpretations are hypothesis-generating and cannot establish specific neurocognitive or neurobiological mechanisms. A more comprehensive analysis incorporating these factors and methods may provide a more accurate understanding of these associations and should be aimed for in future studies. Finally, due to the cross-sectional design, no conclusions can be drawn regarding causality or temporal ordering. The observed associations may reflect correlates, shared vulnerabilities, bidirectional relationships, potential antecedents, or possible consequences. Therefore, future longitudinal studies are needed to clarify the temporal relationships between these variables and orthorexic tendencies.

## 6 Conclusion and outlook

This study examined a range of factors potentially associated with orthorexic tendencies, emphasizing psychological, behavioral, social and clinical domains. In the present sample, orthorexic tendencies were most strongly associated with eating disorder psychopathology, while sport participation, health- and fitness-related social media usage and personality factors were also associated with DOS scores. Overall, the findings are consistent with a spectrum-based interpretation of orthorexic tendencies and highlight the need for future research to clarify their diagnostic status within eating disorder psychopathology. Despite these findings, future cross-sectional studies should aim to replicate these findings in larger and more diverse samples and to take additional relevant factors (such as exercise determinants and sports motives) and potential mediators (perceived control) into account, thus potentially removing methodological inconsistencies in the literature. Meticulously controlled longitudinal studies are needed to clarify temporal ordering and to better distinguish associated factors, potential antecedents, and possible consequences of orthorexic tendencies. Finally, systematic reviews and meta-analyses, analyzing studies that use valid diagnostic and assessment tools, could help consolidate the current state of research and promote a more standardized classification of ON in scientific research.

## Ethics approval

The study involving human participants was reviewed and approved by the local ethics committee (#2024-40, Goethe University Frankfurt, GER).

## Consent for publication

The participants provided their informed consent to participate in this study.

## Data availability statement

The data supporting the findings of this study are available from the corresponding author upon reasonable request.

## Author contributions

AMR, SM, and RV drafted, critically reviewed, and revised the manuscript for important intellectual content. All authors contributed to manuscript revision, read, and approved the submitted version.

## Usage of AI

During the preparation of this work, the authors used generative AI limited to linguistic refinement and for general support in improving analytic code. After using AI, the authors reviewed and edited the content as needed and take full responsibility for the content of the published article.

## Funding

This study was not funded.

## Declaration of competing interest

The authors declare that they have no known competing financial interests or personal relationships that could have appeared to influence the work reported in this paper.

## Footnotes

2 *t* = t statistic, *F* = F statistic, *p* = p value, CI = confidence interval, *g* = Hedges' *g*, ω^2^ = omega squared; MI indicates pooled estimates based on multiple imputation.
