# Supplementary material for: From health to harm: Cross-sectional analysis of factors associated with orthorexia nervosa
Source: Neurosci Appl. 2026 May 12;5:107011. doi: 10.1016/j.nsa.2026.107011 (PMC13197788; doi:10.1016/j.nsa.2026.107011)
Supplement: Supplementary material 1 [file mmc1.docx]

**Supplementary Material**

**Figure A.1**

Forest Plot of the final hierarchical regression model


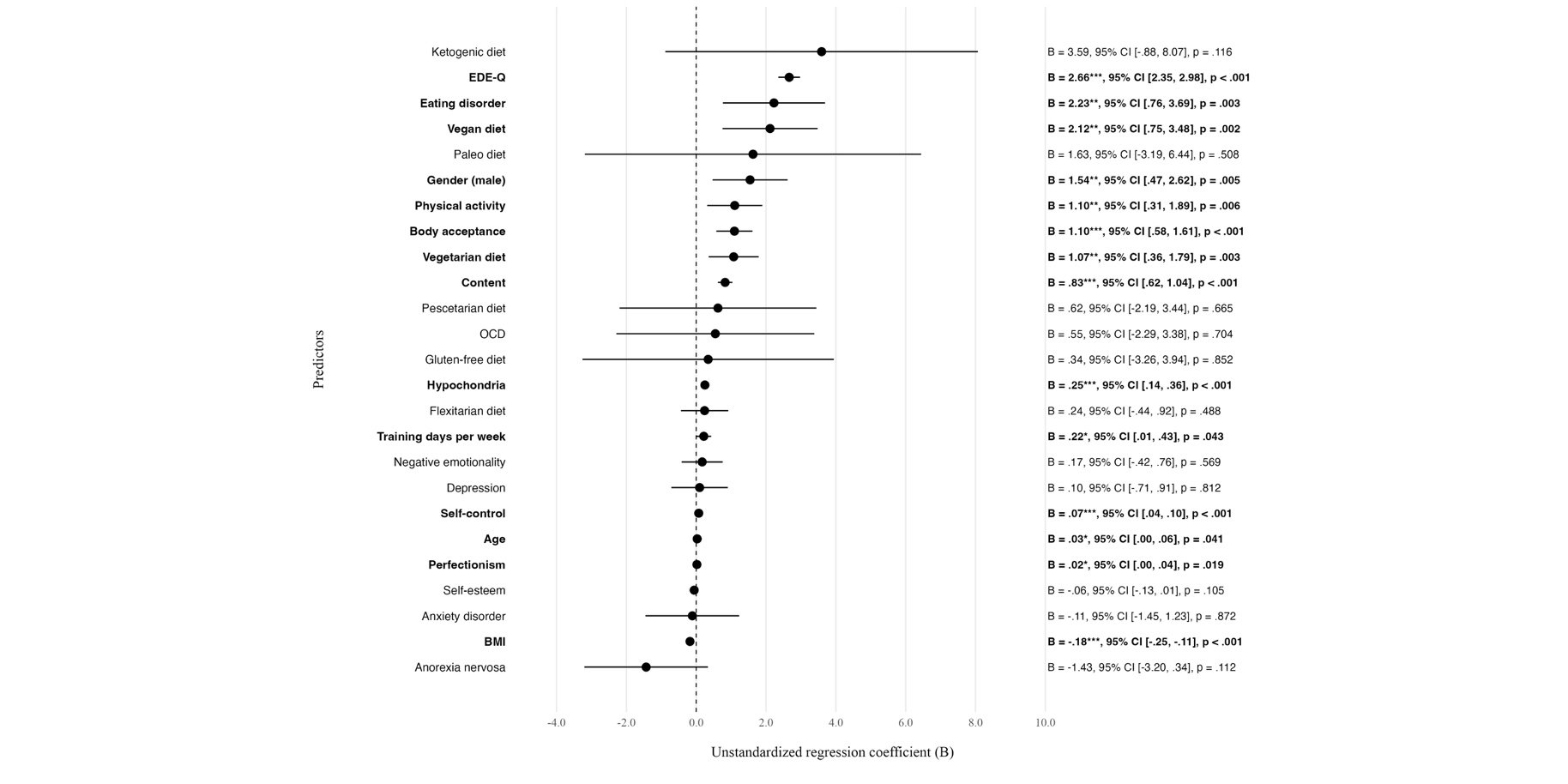


*Notes.* B = unstandardized regression coefficient, CI = confidence interval; significant predictors are displayed in bold; ^*^*p* < .05, ^**^*p* < .01, ^***^*p* < .001. All depicted values are based on imputed data.

**Table A.1**

Correlations among study variables

| **Variable** | **1** | **2** | **3** | **4** | **5** | **6** | **7** | **8** | **9** | **10** | **11** | **12** | **13** | **14** | **15** | **16** | **17** | **18** | **19** | **20** | **21** | **22** |
| --- | --- | --- | --- | --- | --- | --- | --- | --- | --- | --- | --- | --- | --- | --- | --- | --- | --- | --- | --- | --- | --- | --- |
| 1 |  |  |  |  |  |  |  |  |  |  |  |  |  |  |  |  |  |  |  |  |  |  |
| 2 | .62^***^ |  |  |  |  |  |  |  |  |  |  |  |  |  |  |  |  |  |  |  |  |  |
| 3 | .59^***^ | .82^***^ |  |  |  |  |  |  |  |  |  |  |  |  |  |  |  |  |  |  |  |  |
| 4 | .53^***^ | .94^***^ | .65^***^ |  |  |  |  |  |  |  |  |  |  |  |  |  |  |  |  |  |  |  |
| 5 | .52^***^ | .94^***^ | .65^***^ | .91^***^ |  |  |  |  |  |  |  |  |  |  |  |  |  |  |  |  |  |  |
| 6 | .60^***^ | .88^***^ | .63^***^ | .78^***^ | .79^***^ |  |  |  |  |  |  |  |  |  |  |  |  |  |  |  |  |  |
| 7 | .34^***^ | .31^***^ | .19^***^ | .32^***^ | .28^***^ | .33^***^ |  |  |  |  |  |  |  |  |  |  |  |  |  |  |  |  |
| 8 | .34^***^ | .44^***^ | .25^***^ | .46^***^ | .42^***^ | .46^***^ | .45^***^ |  |  |  |  |  |  |  |  |  |  |  |  |  |  |  |
| 9 | .26^***^ | .32^***^ | .17^***^ | .34^***^ | .31^***^ | .35^***^ | .41^***^ | .88^***^ |  |  |  |  |  |  |  |  |  |  |  |  |  |  |
| 10 | .38^***^ | .49^***^ | .30^***^ | .50^***^ | .46^***^ | .49^***^ | .44^***^ | .87^***^ | .67^***^ |  |  |  |  |  |  |  |  |  |  |  |  |  |
| 11 | .23^***^ | .33^***^ | .19^***^ | .34^***^ | .32^***^ | .34^***^ | .33^***^ | .86^***^ | .65^***^ | .58^***^ |  |  |  |  |  |  |  |  |  |  |  |  |
| 12 | .43^***^ | .44^***^ | .30^***^ | .44^***^ | .41^***^ | .43^***^ | .37^***^ | .50^***^ | .42^***^ | .52^***^ | .36^***^ |  |  |  |  |  |  |  |  |  |  |  |
| 13 | .37^***^ | .27^***^ | .20^***^ | .26^***^ | .26^***^ | .25^***^ | .23^***^ | .23^***^ | .21^***^ | .24^***^ | .16^***^ | .78^***^ |  |  |  |  |  |  |  |  |  |  |
| 14 | .20^***^ | .09^*^ | .12^***^ | .07^*^ | .05 | .05 | .03 | .02 | .06 | .00 | -.00 | .18^***^ | .34^***^ |  |  |  |  |  |  |  |  |  |
| 15 | .41^***^ | .46^***^ | .31^***^ | .46^***^ | .43^***^ | .44^***^ | .34^***^ | .56^***^ | .47^***^ | .57^***^ | .40^***^ | .89^***^ | .66^***^ | .13^***^ |  |  |  |  |  |  |  |  |
| 16 | .27^***^ | .34^***^ | .16^***^ | .36^***^ | .32^***^ | .38^***^ | .34^***^ | .57^***^ | .51^***^ | .57^***^ | .40^***^ | .68^***^ | .39^***^ | -.02 | .64^***^ |  |  |  |  |  |  |  |
| 17 | .24^***^ | .25^***^ | .19^***^ | .24^***^ | .23^***^ | .25^***^ | .22^***^ | .23^***^ | .18^***^ | .24^***^ | .17^***^ | .75^***^ | .45^***^ | .10^**^ | .47^***^ | .34^***^ |  |  |  |  |  |  |
| 18 | .30^***^ | .33^***^ | .25^***^ | .32^***^ | .31^***^ | .31^***^ | .28^***^ | .33^***^ | .25^***^ | .36^***^ | .25^***^ | .72^***^ | .34^***^ | .07^*^ | .47^***^ | .38^***^ | .77^***^ |  |  |  |  |  |
| 19 | -.41^***^ | -.75^***^ | -.47^***^ | -.80^***^ | -.76^***^ | -.64^***^ | -.34^***^ | -.53^***^ | -.41^***^ | -.59^***^ | -.37^***^ | -.42^***^ | -.21^***^ | -.04 | -.45^***^ | -.39^***^ | -.21^***^ | -.30^***^ |  |  |  |  |
| 20 | -.41^***^ | -.55^***^ | -.34^***^ | -.56^***^ | -.52^***^ | -.55^***^ | -.34^***^ | -.71^***^ | -.56^***^ | -.78^***^ | -.49^***^ | -.59^***^ | -.29^***^ | -.00 | -.67^***^ | -.63^***^ | -.26^***^ | -.38^***^ | .63^***^ |  |  |  |
| 21 | .11^**^ | -.08^*^ | .01 | -.10^**^ | -.09^**^ | -.08^**^ | -.16^***^ | -.18^***^ | -.12^***^ | -.16^***^ | -.19^***^ | -.01 | .22^***^ | .53^***^ | -.02 | -.28^***^ | -.00 | -.07^*^ | .10^**^ | .15^***^ |  |  |
| 22 | .35^***^ | .17^***^ | .20^***^ | .15^***^ | .14^***^ | .12^***^ | .19^***^ | .05 | .05 | .03 | .04 | .13^***^ | .16^***^ | .14^***^ | .08^**^ | .05 | .08^**^ | .13^***^ | -.08^**^ | .00 | .07^*^ |  |

*Notes.* 1 = DOS, 2 = EDE-Q global score, 3 = EDE-Q Restraint, 4 = EDE-Q Shape Concern, 5 = EDE-Q Weight Concern, 6 = EDE-Q Eating Concern, 7 = Hypochondria, 8 = Negative Emotionality total score, 9 = Negative Emotionality facet Anxiety, 10 = Negative Emotionality facet Depression, 11 = Negative Emotionality facet Emotional Volatility, 12 = Perfectionism global score, 13 = Perfectionism dimension Personal Standards, 14 = Perfectionism dimension Organization, 15 = Perfectionism dimension Concern over Mistakes, 16 = Perfectionism dimension Doubts about Actions, 17 = Perfectionism dimension Parental Expectations, 18 = Perfectionism dimension Parental Criticism, 19 = Body Acceptance, 20 = Self-esteem, 21 = Self-control, 22 = Engagement with health and fitness content on social media; ^*^ *p* < .05. ^**^ *p* < .01. ^***^ *p* < .001. All depicted values are based on imputed data.

**Table A.2**

Partial correlations with orthorexic tendencies controlling for eating disorder symptoms

| **Variable** | ***r*** | **95% CI** | ***p*** |
| --- | --- | --- | --- |
| Hypochondria | .19 | [.13, .25] | < .001 |
| Negative Emotionality | .09 | [.02, .15] | .008 |
| Perfectionism | .22 | [.16, .28] | < .001 |
| Body acceptance | .10 | [.04, .16] | < .001 |
| Self-esteem | -.10 | [-.16, -.04] | .001 |
| Self-control | .19 | [.13, .26] | < .001 |
| Engagement with health and fitness content on social media | .32 | [.26, .37] | < .001 |

*Notes.* *r* = Partial correlations between DOS scores and each predictor while controlling for eating disorder symptoms (EDE-Q global score), CI = confidence interval. All depicted values are based on imputed data.
